# CpWRKY50 drives jasmonic acid defense in boosting papaya's resistance to anthracnose

**DOI:** 10.1093/plphys/kiae521

**Published:** 2024-10-07

**Authors:** Ritu Singh

**Affiliations:** Plant Physiology, American Society of Plant Biologists; Department of Plant Science, University of California, Davis, CA 95616, USA

Papaya (*Carica papaya* L.) is a tropical and subtropical fruit renowned for its high nutritional and medicinal value. However, the sustainability of papaya-related industries is increasingly at risk due to anthracnose, a severe postharvest disease caused by the hemibiotrophic fungus *Colletotrichum brevisporum*, which significantly impacts the quality and yield of papaya fruit (Zhang et al. 2021; Yang et al. 2024a, 2024b). Developing genetic resistance to anthracnose has become a critical breeding objective for improving papaya cultivars. To implement this, it is crucial to identify disease resistance–related genes and study their molecular mechanisms in papaya.

Plant defense mechanisms are regulated by various signaling pathways, including oxidative bursts, mitogen-activated protein kinase cascades, transcription factors (TFs), and phytohormones. *WRKY* TFs are one of the largest families of transcriptional regulators in plants and are integral components of defense signaling networks (Javed and Gao 2023). A recent study published in *Plant Physiology* by Yang et al. (2024a, 2024b) identified a differentially expressed *CpWRKY50* gene based on previous transcriptomic data from papaya infected with *C. brevisporum* (Yang et al. 2022). *CpWRKY50* was highly upregulated in the anthracnose-resistant papaya “Zhufeng,” than in anthracnose-susceptible “Y61.” Further testing across other papaya cultivars revealed that all tested anthracnose-resistant cultivars exhibited higher expression of *CpWRKY50* than the susceptible ones, suggesting that *CpWRKY50* plays a significant role in resistance to anthracnose.

To characterize the function of the CpWRKY50 protein, Yang and coworkers assessed its ability to activate or repress transcription. They transformed yeast cells with vectors containing full-length or truncated *CpWRKY50* fragments fused to the GAL4 DNA-binding domain. The full-length CpWRKY50 protein showed transactivation activity, as indicated by the growth of the transformants on selective media. In contrast, N- and C-terminal fragments failed to grow on selective media. These data suggest that CpWRKY50 has transactivation activity and both termini are essential for transactivation.

Previous studies have demonstrated that *WRKY* play complex roles in disease resistance by regulating jasmonic acid (JA) and salicylic acid (SA) pathways (Chen et al. 2021). Yang et al. (2024a, 2024b) observed that *CpWRKY50* transcript levels increased ∼8.5 fold after treatment with methyl jasmonate (MeJA, a stable derivative of JA), while SA treatment resulted in a modest decrease in *CpWRKY50* expression. The authors also cultured *C. brevisporum* mycelia on potato dextrose agar plates containing different concentrations of MeJA and found that MeJA treatment inhibited the growth of *C. brevisporum* in a concentration-dependent manner. Additionally, exogenous MeJA treatment effectively slowed *C. brevisporum* diffusion in papaya fruits, as non–MeJA-treated papaya fruits showed significant anthracnose plaques, whereas MeJA-treated fruits had no visible or smaller plaques. These findings suggest that *CpWRKY50* may participate in anthracnose disease resistance by regulating the JA-mediated signaling pathway.

To further investigate the role of *CpWRKY50* in papaya anthracnose resistance, Yang et al. (2024a, 2024b) performed *Agrobacterium*-mediated transient overexpression (OE) of *CpWRKY50* in fruits of the anthracnose-susceptible cultivar, along with an empty vector as a control. Fruits transformed with the empty vector had more and larger-sized plaques than the *OE-CpWRKY50*, indicating a positive role of *CpWRKY50* in *C. brevisporum* resistance. To confirm these results, *CpWRKY50* was also overexpressed in tomatoes, where disease onset was delayed in the transgenic lines compared to the wild type. Furthermore, JA and JA-Ile levels were also higher in the transgenic tomatoes both without and with *C. brevisporum* infection. These results indicated that increased expression of *CpWRKY50* leads to higher JA content and enhances anthracnose resistance.

The WRKY family can directly bind to the conserved motif (TTGACC) in target gene promoters and regulate their transcription. Using DNA affinity purification sequencing, Yang et al. (2024a, 2024b) identified several genes involved in the JA pathway that are likely regulated by *CpWRKY50*, including *CpMYC2*, *CpJAZ1*, *CpJAR1*, and *CpPR4*. Yeast 1-hybrid assays and electrophoretic mobility shift assays confirmed that CpWRKY50 binds to W-box motifs (TTGACC) in the promoters of *CpMYC2* and *CpPR4*. Transient dual-luciferase assays in *Nicotiana benthamiana* leaves showed that CpWRKY50 positively activates the expression of *CpMYC2* and *CpPR4*. Next, the authors examined *CpMYC2* and *CpPR4* expression in transient *CpWRKY50-*overexpressed papaya fruits and tomatoes. *CpMYC2* and *CpPR4* expression levels were higher in both overexpressed papaya fruits and tomatoes compared with controls. Additionally, the expression levels of *CpMYC2* and *CpPR4* in anthracnose-resistant papaya “Zhufeng” were significantly higher than that of anthracnose-susceptible papaya “Y61.” These results indicate that CpWRKY50 can bind to the promoters of *CpMYC2* and *CpPR4*, positively activating their expression.

**Figure 1.**
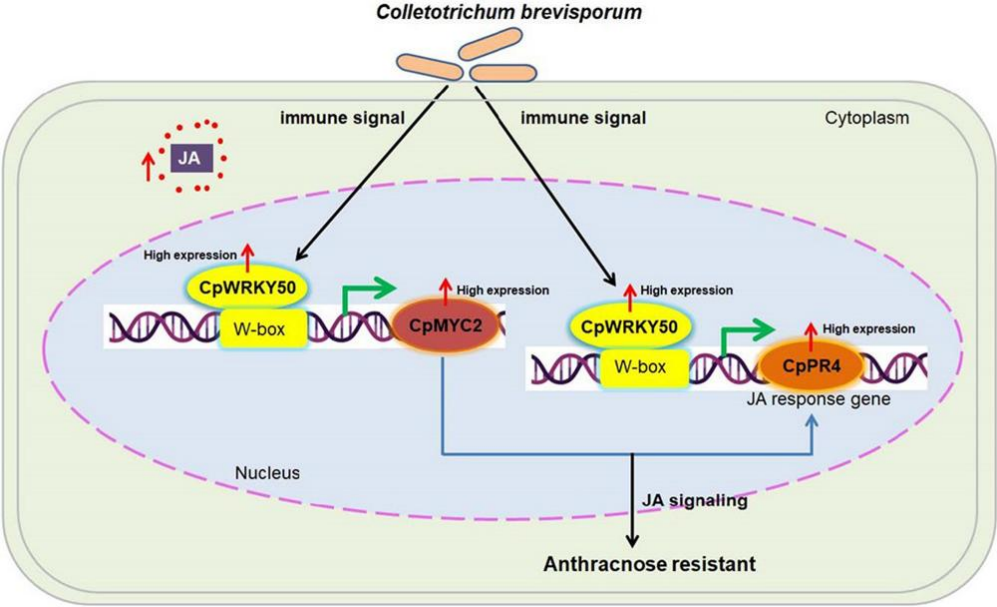
A proposed model for CpWRKY50 regulation in papaya anthracnose-resistant cultivar to *C. brevisporum* infection (adapted from Figure 8 of Yang et al. 2024a, 2024b). *C. brevisporum* infection increases the expression of CpWRKY50, which then binds to the W-box motifs in the promoters of *CpMYC2* and *CpPR4*, leading to their elevated expression. This results in activation of JA signalling pathways, causing increased JA levels and enhanced anthracnose resistance in papaya.

Overall, Yang et al. (2024a, 2024b) observed that *CpWRKY50* positively regulates anthracnose resistance in papaya by modulating the JA signaling pathway. In anthracnose-resistant cultivars, *CpWRKY50* is upregulated in response to *C. brevisporum* infection. *CpWRKY50* acts as a transcriptional activator, enhancing JA content and activating the expression of *CpMYC2* and *CpPR4* by binding to W-box motifs in their promoters, thereby enhancing resistance to papaya anthracnose (Fig. 1).
